# Using curcumin to prevent structural and behavioral changes of medial prefrontal cortex induced by sleep deprivation in rats

**DOI:** 10.17179/excli2017-139

**Published:** 2017-04-18

**Authors:** Ali Noorafshan, Fatemeh Karimi, Saied Karbalay-Doust, Ali Mohammad Kamali

**Affiliations:** 1 Histomorphometry and Stereology Research Center, Shiraz University of Medical Sciences, Shiraz, Iran; 2 Anatomy Department, School of Medicine, Shiraz University of Medical Sciences, Shiraz, Iran; 3 Department of Neuroscience, School of Advanced Medical Sciences and Technologies, Shiraz University of Medical Sciences, Shiraz, Iran

**Keywords:** sleep deprivation, mPFC, curcumin, stereology, rat

## Abstract

Sleep Deprivation (SD) is known to result in a range of neurological consequences in chronically-afflicted subjects. Curcumin, a natural substance, has neuroprotective properties. This study aimed to evaluate the effects of curcumin on the medial Prefrontal Cortex (mPFC) of SD rats. Male rats were arbitrarily assigned to nine groups, including control, curcumin (100 mg/kg/day), olive oil, SD, SD+curcumin, SD+olive oil, grid, grid+curcumin, and grid+olive oil groups. SD was induced by a multiplatform box containing water. After a period of 21 days, the learning and memory of the rats were tested in an eight-arm radial maze. Afterwards, their brains were evaluated using stereological methods. Concomitant treatment of curcumin during SD caused fewer errors during evaluation of the working and reference memory errors in the acquisition and retention phases. The overall volume of the mPFC, Infralimbic Cortex (ILC), Prelimbic Cortex (PLC), Anterior Cingulate Cortex (ACC) and the total number of neurons and glial cells reduced by 20 %-40 % on average in the SD animals in comparison to the control group. This indicated atrophic changes and cell loss in these areas (p < 0.01). The dendrites' length and the number of spines per dendrite also reduced by 35 %-55 % in the SD rats compared to the ones in the control group (p < 0.01). Yet, treatment of the SD animals with curcumin prevented the atrophic changes of the mPFC, cell loss, and dendritic changes (p < 0.05). SD induced structural changes in the mPFC and memory impairment in the rats. However, curcumin could protect their PFC.

## Introduction

Sleep, as an active and cyclic behavior, serves diverse functions, such as growth, renovation, and learning/memory consolidation and restoration. In today's hectic life, disorderly lifestyle, psychological and emotional disturbances as well as aging have led to Sleep Deprivation (SD) and its neurological consequences (Mishra et al., 2016[18]). Previous studies have shown that SD could result in such problems as mood alterations, mental injuries, troubled performance, and cognitive disturbances (Motomura et al., 2013[19]; Carskadon, 2011[3]). Quite a few animal studies have also focused on the effects of SD on different brain regions. However, the link between SD and prefrontal cortex has not been thoroughly investigated. The medial Prefrontal Cortex (mPFC) is one of the areas that receive important innervations from the brain stem and hippocampus. This area also plays a fundamental role in memory and learning. The mPFC of rats is generally subdivided into three cytoarchitectonic parts, including Infralimbic Cortex (ILC), Prelimbic Cortex (PLC), and Anterior Cingulate Cortex (ACC) (Pezze et al., 2016[22]). There is scientific evidence that the mPFC is responsible for efficient operation of a number of cognitive functions, such as attention, memory, and behavioral flexibility. The mPFC also mediates cognitive behavior (Granon and Poucet, 2000[8]), which is vulnerable to the disruption caused by SD. Other PFC-mediated cognitive tasks such as memory, planning, decision-making are affected by SD (Wu et al., 2006[30]). Therefore, the present research was designed to fulfill several goals. The main aim of this study was to evaluate the effects of chronic SD on the mPFC structure using stereological methods. The second goal is to find a protective factor to be consumed in case of SD. Curcumin (CUR) is the major curcuminoid of turmeric, which belongs to a member of the ginger family. CUR is known to have a variety of neuroprotective properties (Weber et al., 2005[28]). CUR has been evaluated in a number of neurological diseases, such as Parkinson's disease (Zbarsky et al., 2005[31]), cerebral injury (Ghoneim et al., 2002[7]) and age-associated neurodegeneration (Calabrese et al., 2003[2]). Considering the availability of turmeric and its neuroprotective prosperities, CUR was considered as the agent to be evaluated after SD. This research was conducted on a rat model of SD to find answers to the following questions:

1. Does SD influence rats' spatial memory and learning?
2. Does SD have any effects on the volume of the mPFC and its ACC, PLC, and ILC?
3. Does the number of neurons and glial cells in the mPFC change after SD?
4. Do the dendrites' length and their spines' morphology (mushroom, thin, and stubby types) undergo any change after SD?
5. Can CUR protect the alteration in spatial memory, learning and mPFC structure of the SD animals?

To determine the structural changes in mPFC, the tissue was examined using stereological techniques.

## Materials and Methods

### Animals

A total of 63 adult male Sprague-Dawley rats aged 3 months (250-300 g) were obtained from the Animal Laboratory Center of the University. The animal trial was approved by the Ethics Committee of the University (approval No. 94-7624) and all manipulations were done under the regular instructions of the Animal Ethics Committee. First, the rats were arbitrarily divided into nine groups each containing 7 animals. The animals were then kept in a room with normal conditions and temperature (22-24 °C) and had free access to water and food. Through daily gavage feeding, the groups received the following:

- Group I: (Control) 1.5 ml of distilled water
- Group II: Curcumin (100 mg/kg/day) dissolved in olive oil
- Group III: 1.5 ml of olive oil
- Group IV: Distilled water
- Group V: Curcumin
- Group VI: Olive oil
- Groups VII: Distilled water
- Group VIII: Curcumin
- Group IX: Olive oil

It should also be noted that groups IV, V and VI experienced SD and the animals in the last three groups were placed on cages with grid floors. After a period of 21 days for the SD protocol, the rats underwent the eight-arm radial maze test and were then sacrificed. 

### Sleep deprivation procedure

The setup used in this study was a modified multi-platform container made of plastic that have been explained earlier (Kamali et al., 2016[12]). The container was then filled with water. When a rat entered the sleep, it touched the water by tilting its head downwards or fell into the water due to muscle atonia. The SD period continued for 21 days during which the rats were kept in the multiple-platform box for 18 hours from 6:00 P.M. to 12:00 noon on the subsequent day. During the next 6 hours, the rats were permitted to fall asleep (12:00 noon to 6:00 pm). The light schedule of the SD-boxes, on from 6:00 A.M. until 6:00 P.M, was in accordance with the light/dark cycle similar to the non-SD animals (Wang et al., 2014[27]). In the grid groups (VII, VIII, IX), the rats were located on wire-mesh grids in the deprivation container. The grid was made of stainless steel. This was incorporated as a control group for SD. Finally, the rats in these groups also underwent the eight-arm radial maze test. 

### Assessment of behavior in the eight-arm radial maze

To assess spatial learning and memory, the eight-arm radial maze test was carried out according to the previous study (Karkada et al., 2012[13]). The first stage (also known as the adaptation phase) in which the rats were allowed to discover the baited arms of the maze for 10 min lasted for two consecutive days. In the second phase (also known as the acquisition phase), the rats were given two five-minute trials per day until they reached the learning standards which was defined as reaching 80 % correct choices; i.e., at least four correct entries out of five. This period lasted for eight to fifteen days. The third stage (also known as the retention period) started ten days after the acquisition phase. The average of the two trials and the percentage of correct choices and errors were used for analysis.

### Tissue preparation 

**Figure 1:**
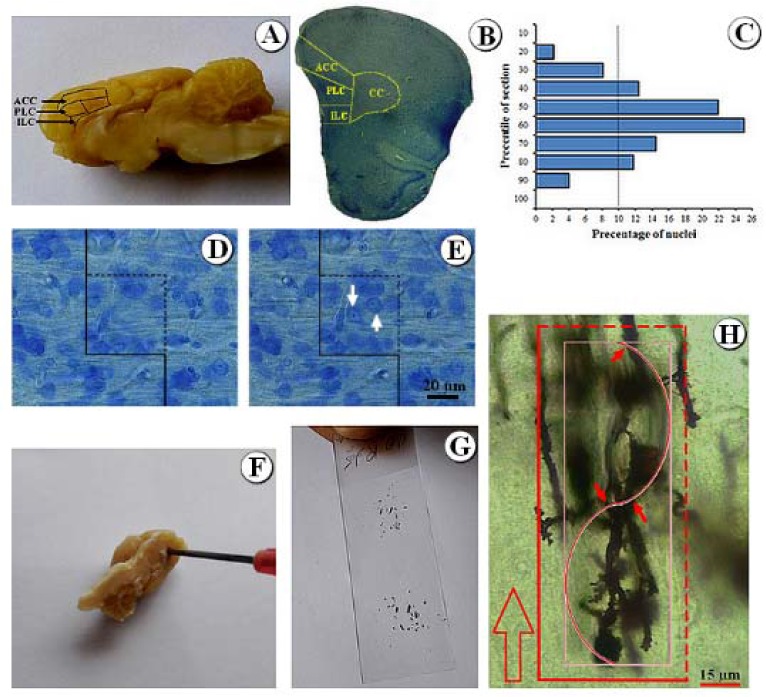
Stereological methods. [A] mPFC and its anterior cingulate cortex (ACC), prelimbic (PLC), and infralimbic (ILC) subdivisions. [B] Stereological methods. Subdivisions of them PFC were traced and the sum of the area of each structure was determined using the software. [C] The Z-axis distribution of the nuclei was plotted to define the disector's height. There are ten columns each representing the percent of the counted nuclei in ten percent of the section thickness from the top to the bottom of the section. [D, E] Optical disector method was applied to count the cells. The cells whose nucleoli came into focus during scanning the disector's height and did not touch the left and bottom borders of the frame were counted (arrows). [F] Vertical uniform random sectioning. The vertical cylinders were punched out from the mPFC cortex vertical to its pial surface. [G] The cylinders were sectioned using a microtome and mounted on a slide. [H] When the sections were scanned, the number of cell bodies of the neurons was counted using the optical disector method and unbiased counting frame. The total number of intersections between the dendrite axes and the cycloid was counted (small arrows). The cycloid was positioned parallel to the vertical axis (large arrow).

The animals were anesthetized and their brains were removed. Then, the right hemispheres were processed, coronally sectioned (26 μm thick) and stained using Giemsa and were used to estimate the number of neurons and glial cells as well as the volume of the mPFC. Besides, the slabs of the left hemispheres were processed and stained with Golgi impregnation method (Figure 1(Fig. 1)).

### Estimation of the volume 

Using a stereomicroscope, the live image of each brain section was evaluated according to the rat brain atlas. The volume *"V(mPFC)"* was estimated using the Cavalieri method (Kristiansen and Nyengaard, 2012[14]). The sum of the area of the favored structure (ΣA) (mPFC, ACC, PLC, and ILC) was estimated using the software designed at the University. The distance between the sampled sections was defined as “d”. Finally, the volume of each structure was estimated by the following formula:


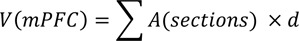


### Estimation of the number 

A videomicroscopy system along with an oil immersion lens (40×, numerical aperture: 1.3) were used in order to estimate the total number of the mPFC neurons and glial cells according to the optical disector method. To analyze the appropriate guard zone and the height of the disector (h), Z-axis distribution of the nuclei was plotted (Figure 1(Fig. 1)). The total number of the neurons was estimated by multiplying the numerical density” 


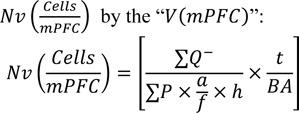


where "Σ*Q*^-^" is the number of the nuclei coming into focus during the scanning of “*h*” (the height of the disector). "Σ*P*" is the total number of counting frames in all fields, "a/f" is the frame area, “*t*” is the mean section thickness measured in every sampled field using the microcator (20 µm on average), and “*BA*” is the block advance of the microtome (Rubinow and Juraska, 2009[24]).

### Estimation of the coefficient of error (CE)

After the cross-sectional areas “ΣA” were estimated by the software, CE (V) was calculated using the following formula:


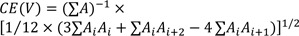


The CE for the estimate of the total number of neurons and glial cells, CE (*N*), was derived from *CE(V)* and *CE(Nv)* using the following formula (Gundersen et al.,1999[9]):


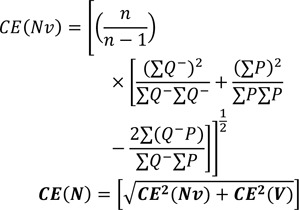


### Estimation of the length of dendrites and spine morphology

Length estimation was performed on 9-10 vertical uniform random cylinders (Figure 1(Fig. 1)). 100 μm thickness slabs were obtained and stained with Golgi method (De Ruiter and Uylings, 1987[6]). The mean dendritic length per neuron was calculated by dividing the total length by the total number of neurons. To estimate the length, a cycloid grid and a counting frame were mounted on the live images of the mPFC parallel to the vertical axis of the cylinder. Using a videomicroscopy system equipped with a high numerical aperture objective lens, the slab height was scanned. Finally the following formula was used:


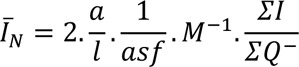


where "a/l" is the test area per cycloid test length, *"asf"* is the area associated with the cycloid grid divided by the area of the counting frame, and “*M*” is the final magnification at ×4000, *"ΣI"* is the total number of intersections, "*ΣQ*^-^" is the total number of neuron bodies. To estimate the density and morphology of the spines, the above-mentioned dendrites were examined. Dendritic spines were identified and classified as stubby, thin or mushroom spines (Chapleau et al., 2008[4]).

### Statistical analysis

The data were analyzed using Kruskall-Wallis, Mann-Whitney U-test, and either two-way or one-way ANOVA followed by Tukey's post-hoc test. Besides, p≤0.05 was considered to be statistically significant.

## Results

### Correct choices during the acquisition phase

**Figure 2:**
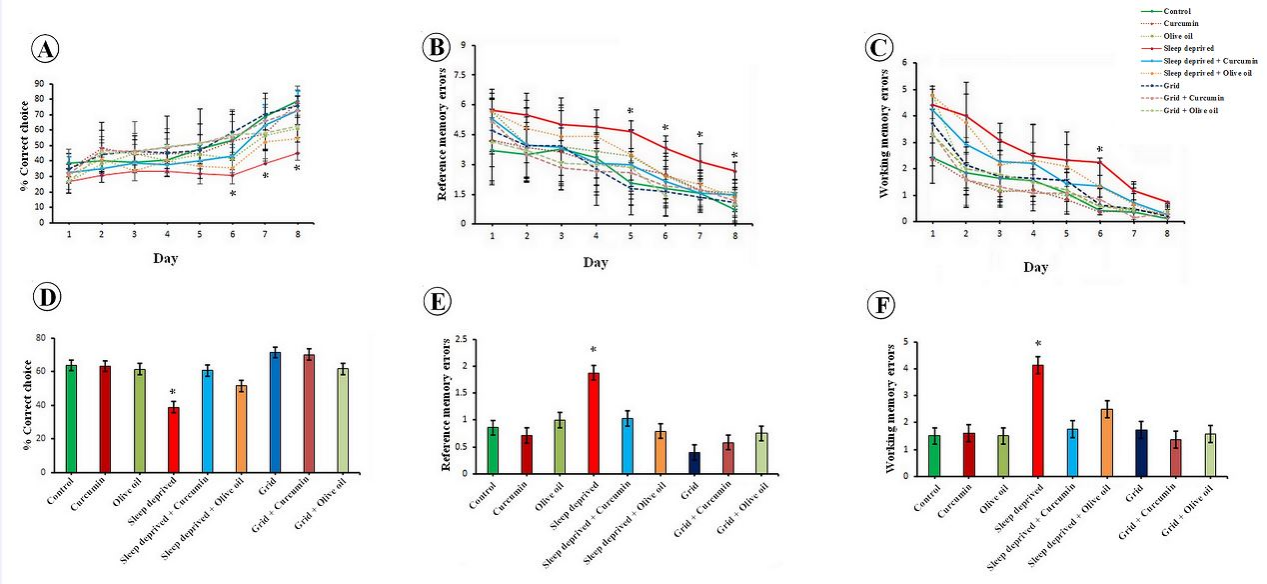
Eight-arm radial maze evaluations. [A] Mean±SD of the percentage of correct choices during the acquisition session. [B] Mean±SD of the number of reference memory errors during the acquisition session. [C] Mean±SD of the number of working memory errors during the acquisition session. [D] Comparison of the mean±SD of the percentage of correct choices during the retention session. *P < 0.001, SD vs. SD+curcumin or control. [E] Comparison of the mean±SD of the number of reference memory errors during the retention session. *P < 0.01, SD vs. SD+curcumin or control. [F] Comparison of the mean±SD of the number of working memory errors during the retention session. *P < 0.01, SD vs. SD+curcumin or control.

On the first three days, all animals exhibited similar learning performances in the radial maze test, while they seemed to gradually improve their ability to learn the location of the baited arm during the learning phase from the 4^th^ to the 8^th^ day (Figure 2(Fig. 2)). A two-way repeated measure ANOVA was performed with “training days” as the within-subjects factor and “experimental groups” as the between-subjects factor. A significant difference was found for “training days” [(p < 0.001); F(7,378)=85.92)], “experimental groups” [(p < 0.001); F(8,54)=8.11)], and the “training days” by experimental groups” interaction [(p < 0.01); F(56,378)= 1.75)]. This suggests that the performance of the animals changed during the acquisition and that this change in performance was different between the groups (p < 0.001). The rats in the SD group showed less progress in the selection of the correct choices compared to the control animals from the 1^st ^ to 8^th ^ day.

The score of the correct choices increased in the rats of the SD+curcumin group in comparison to the SD animals (p < 0.001). This demonstrated that concomitant treatment of curcumin during SD prevented the reduction of scores of the correct choices in the acquisition session.

### Working and reference memory errors during the acquisition phase

A two-way repeated measures' ANOVA was performed to evaluate working and reference memory errors with the “acquisition days” as the within-subjects factor and “experimental groups” as the between-subjects factor. A significant effect was observed for reference memory error [p < 0.001), F(7,378) =64.19], “experimental groups” [p < 0.01), F(8,54)=4.00], and the “acquisition days” by “experimental groups” interaction [p < 0.01), F (56,378) =1.51]. The test also showed a significant effect on the working memory error [p < 0.001), F(7,378) =76.20], “experimental groups” [p < 0.01), F8,54)=4.26], and the “acquisition days” by “experimental groups” interaction [p < 0.03), F(56,378)=1.46]. The data showed that both working and reference memory errors during the acquisition phase changed compared to the days of training which were different among the study groups (Figure 2(Fig. 2)). The rats in the SD groups showed more memory errors (working and reference) compared to the control and grid animals. Fewer errors were observed for the rats of SD+curcumin group in comparison to the SD animals (p < 0.03). This demonstrated that concomitant treatment of curcumin during SD caused fewer errors during evaluation of the working and reference memories in the acquisition session. 

### Correct choices during retention testing

The one-way ANOVA was used to analyze the percentage of correct choices during the retention session. The results demonstrated a significant difference among the study groups in this respect [(p < 0.001); F(8,54)=9.28] (Figure 2(Fig. 2)). Furthermore, the SD group revealed a significant reduction in the percentage of correct choices compared to the control group (p < 0.001). Nonetheless, concomitant treatment with curcumin during SD prevented the reduction in the scores of correct choices in the retention session.

### Reference memory errors and working memory errors during retention testing

A significant difference was observed among the groups regarding the number of reference memory errors [(p < 0.01); F(8,54) =3.11], and working memory errors [(p < 0.001); F(8,54)=6.08]. Besides, the rats in the SD group showed more errors during the retention testing in comparison to the control rats (p < 0.01) (Figure 2(Fig. 2)). However, concomitant treatment with curcumin during SD led to fewer errors during the evaluation of reference and working memories in the retention session (Figure 2(Fig. 2)). 

### Volumes of the structures

**Figure 3:**
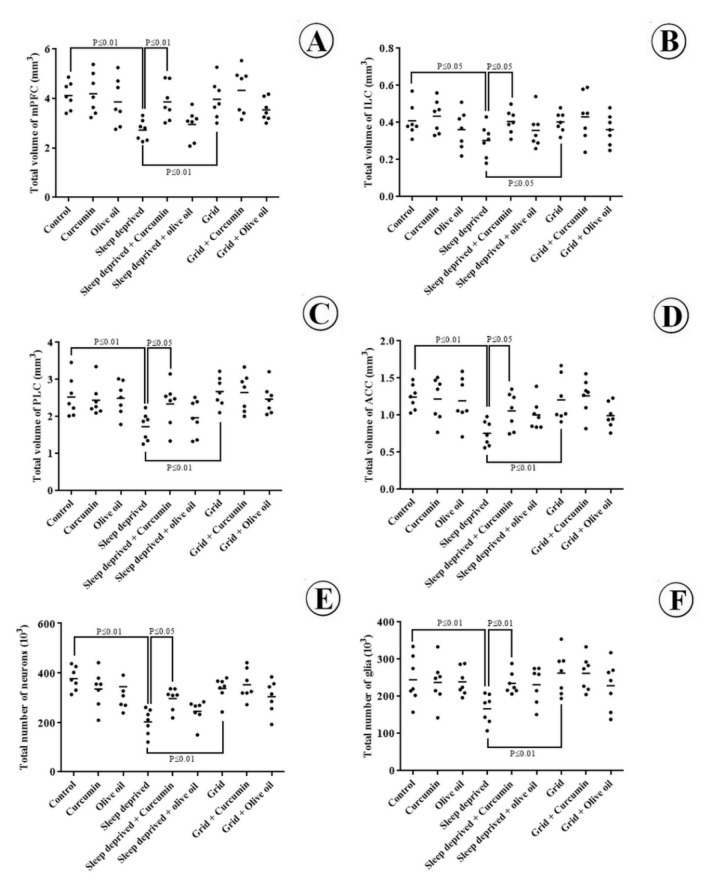
The volume of the cortices and number of neurons and glial cells. [A] Dot plots showing the total volume of mPFC, [B] ILC, [C] PLC, and [D] ACC. [E] Total number of the neuronal cells and [F] glial cells of mPFC. Each dot represents an animal in the SD group with or without curcumin treatment. The horizontal bars show the means of the parameters in the study groups. P-values have been indicated.

The data are presented in Figure 3(Fig. 3). Accordingly, the total volume of the mPFC, ACC, PLC, and ILC reduced by 34, 39, 32 and 27 % respectively in the SD animals in comparison to the control group (p < 0.05) which indicated atrophic changes in these areas. Nevertheless, treatment of the SD animals with curcumin prevented the atrophic changes in the mPFC (and its subregions) compared to the SD group (p < 0.05). The results revealed no statistically significant changes in the volumes of the mPFC, ACC, PLC, and ILC in the experimental groups compared to the control animals. CE of the volume estimation using the Cavalieri method was 0.01-0.02. 

### Total number of cells

The data related to the cell population are presented in Figure 3(Fig. 3). As the figure depicts, the total number of neurons and glial cells of the mPFC reduced by 36 % and 31 % respectively in the SD animals in comparison to the control group (p < 0.01). The number of neurons also reduced, but less considerably, in the SD+curcumin animals as compared to the SD group (p < 0.05). Yet, treatment of the SD animals with curcumin prevented glial cells loss in the mPFC compared to the SD group (p < 0.01). The results revealed no statistically significant changes in the cell population (neurons or glia) in the experimental groups as compared to the control groups. CE of the number estimation using the optical disector method was 0.04-0.05. 

### Total length of dendrites per neuron

**Figure 4:**
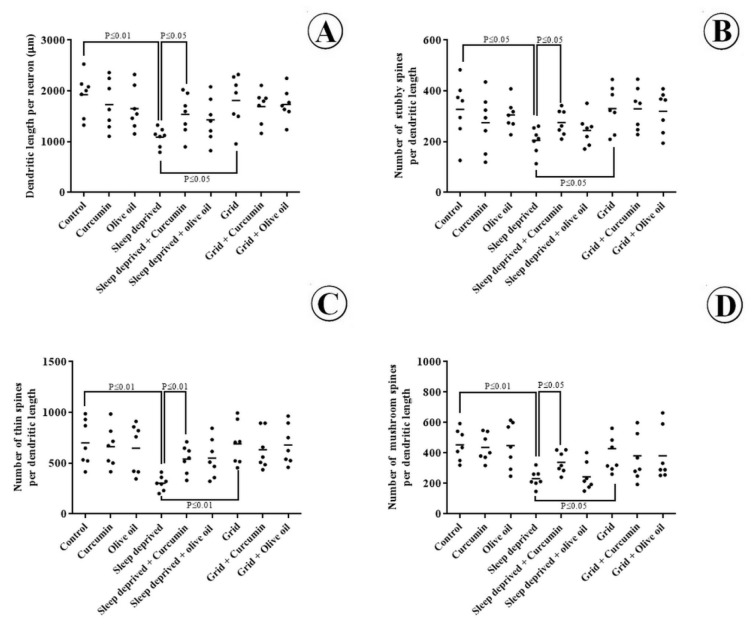
Dendrites' length and spines' morphology evaluation. [A] Total length of the dendrites per neuron in different groups, including control, olive oil, curcumin, SD, SD+curcumin, SD+olive oil, grid, grid+curcumin, and grid+olive oil. [B] Density of the dendritic spines. The density of mushroom spines per neuron has been shown in the mPFC of different groups. A significant difference has been indicated between SD and other groups (P < 0.05). [C] The density of thin spines per neuron has been shown in the mPFC of different groups. A significant difference has been revealed between SD and other groups (P < 0.01). [D] The density of stubby spines per neuron has been shown in the mPFC of different groups. A significant difference has been indicated between SD and other groups (P < 0.05). Each dot represents an animal in different groups. The horizontal bars show the means of the parameters in the study groups. P-values have been indicated.

The data related to dendrites length are presented in Figure 4(Fig. 4). The results showed that the mean dendrites' length of the mPFC was reduced by 43 % in the SD group in comparison to the control group (p < 0.01). The dendrites length also reduced less substantially in the SD+curcumin animals in comparison to the SD group (p < 0.05). No statistically significant changes were identified in the mean dendrites length per neuron in the experimental groups compared to the control group.

### Density and morphology of spines

The results showed that the mean number of stubby, thin, and mushroom spines per dendrite length reduced by 37 %, 54 % and 49 % respectively in the SD group in comparison to the control group (p < 0.05; Figure 4(Fig. 4)). Yet, concomitant treatment of SD+curcumin prevented the loss of dendritic spines in the mPFC. No statistically significant changes were seen in the mean number of dendritic spines in the experimental groups compared to the control group.

### Qualitative evaluation of mPFC

**Figure 5:**
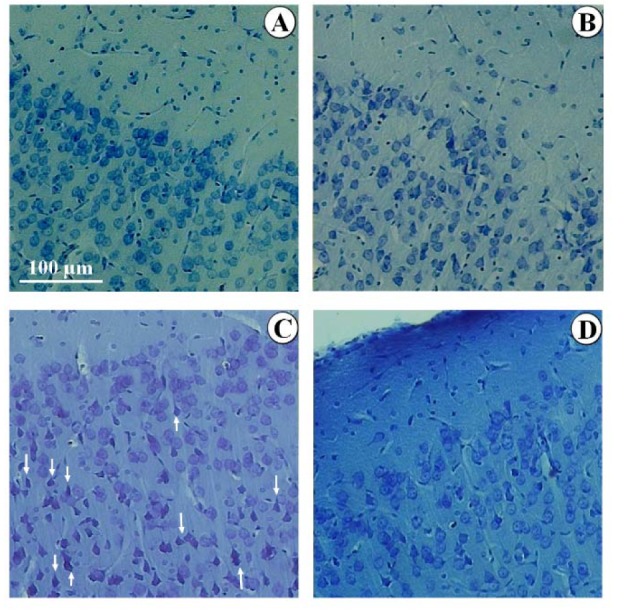
Histological evaluation of the mPFC stained with Giemsa. Evaluation of mPFC revealed a normal appearance in the control [A], curcumin, olive oil, grid+curcumin, and grid+olive oil groups. The grid group has been presented [B]. Lower, shrunk, pyknotic, and smaller granular cells were seen in the SD animals [C] in comparison to the control group. The appearance of mPFC was recovered in the SD+ curcumin animals [D].

Histology of the mPFC is depicted in Figure 5(Fig. 5). Accordingly, there were no significant changes in the histological appearance of the control group compared to other groups, including curcumin, olive oil, grid with curcumin, and grid with olive oil. In addition, the SD animals showed a lower cell population. The remaining cells also seemed shrunk, pyknotic, and smaller in comparison to the control group. These histological changes seemed to have recovered in the SD+curcumin animals. 

## Discussion

The present study investigated the effect of SD on spatial memory and structural changes of the mPFC in a rat model. SD has been considered to be one of the saboteurs in a variety of brain disorders, including memory dysfunction, depression, and psychosis (Mishra et al., 2016[18]). The first part of this study assessed the effects of SD on memory impairment. These impairments could be correlated with neurons and cell loss in the mPFC. This finding accords with the previous studies reporting the effects of SD on the nervous system (Tung et al., 2005[26]). Other researchers have also reported that manipulations of the mPFC could impair memory in eight-arm radial maze, shuttle box and Y-maze tasks (Mehdipour et al., 2015[17]; Lapish et al., 2015[15]). Therefore, the structural changes of the mPFC (reduction in the total volume and loss of neurons and glial cells) could explain this type of memory dysfunction evaluated in the eight-arm radial maze. In line with our study, Abushov (2009[1]) reported that SD reduced the number of neurons, synapses, and behavioral reactions. In addition, Winters et al. (2011[29]) showed that SD altered synaptic and intrinsic neuronal properties in mice prefrontal cortex (Winters et al., 2011[29]). The present survey also revealed volume reduction in the three subdivisions of mPFC. These anatomic changes were correlated to the physiological activity of this region as Chauveau et al. (2014[5]) reported that SD decreased the activity of the prelimbic, infralimbic, and cingulate cortices (Chauveau et al., 2014[5]). It has also been approved that the efficient function of mPFC depended on the integrity of the cortex (Leenaars et al., 2012[16]). 

Despite the evident effects of SD on PFC function, it remains scantily understood how sleep loss affects the essential characteristics of neurons and/or glial cells in the prefrontal cortex. In general, it is assumed that SD raises oxidative stress in diverse brain regions, including the mPFC (Ramanathan et al., 2002[23]). Another mechanism could be clarified by the research by Jiang et al. (2006[11]) who reported that neuron apoptosis could be detected in sleep deprived-animals (Jiang et al., 2006[11]).

Altered activity of neurotransmitters, including monoaminergic and cholinergic, has also been considered to be another underlying factor for the physiological findings related to the mPFC (Leenaars et al., 2012[16]).

The present study also evaluated a neuroprotective agent; i.e., CUR, to avoid the changes in the SD rats. Several researchers have clarified that CUR could recover learning and memory abilities in rats in different conditions. Indeed, Guo et al. (2013[10]) indicated that CUR had a protective effect on HIV-neurotoxicity by reducing microglial inflammation and preventing neuronal apoptosis (Guo et al., 2013[10]). Pan et al. (2008[21]) also reported that CUR could facilitate learning and memory function by diminishing or preventing lipid peroxidation in the brain region of aged rats. In general, CUR is a well-known oxygen free radical scavenger (Pan et al., 2008[21]). Sun et al. (2013[25]) disclosed that CUR could also recover learning and memory in senescence-accelerated mice. Similarly, our previous studies revealed that CUR could play protective roles in rat prefrontal cortical neurons in different pathological conditions (Noorafshan et al., 2015[20]). SD could diminish the spatial memories and affect the mPFC structure. However, CUR could preserve the behavioral and structural changes of the mPFC.

## Acknowledgement

This article was a part of the thesis written by Fatemeh Karimi, PhD candidate of Anatomy. This work was performed at Histomorphometry and Stereology Research Center and was financially supported by grant No. 94-7624 from the University. 

## Conflict of interest

The authors declare no conflict of interest.
